# Consumer food environments change over a 5-year period

**DOI:** 10.1017/S1368980024001721

**Published:** 2024-10-03

**Authors:** Patrícia Pinheiro de Freitas, Mariana Souza Lopes, Mariana Carvalho de Menezes, Patrícia Constante Jaime, Aline Cristine Souza Lopes

**Affiliations:** 1 Universidade Federal dos Vales do Jequitinhonha e Mucuri, Diamantina, Minas Gerais, Brazil; 2 Universidade Federal da Paraíba, João Pessoa, Paraíba, Brazil; 3 Nutrition Department, Universidade Federal de Minas Gerais, Belo Horizonte, Minas Gerais, Brazil; 4 Department of Nutrition, School of Public Health, University of São Paulo, São Paulo, São Paulo, Brazil

**Keywords:** Food environment, Fruit, Vegetable, Ultra-processed food, Longitudinal study

## Abstract

**Objective:**

Evaluate the 5-year changes in the consumers’ food environment in the area of a health promotion service in Brazilian primary health care. Our hypothesis is that the consumers’ food environment in the areas with primary healthcare services has changes that may favour healthy eating habits over time.

**Design:**

Longitudinal study.

**Setting:**

The territory around the primary healthcare services in Belo Horizonte, Minas Gerais, Brazil.

**Participants:**

All food stores and open-air food markets that sell fruits and vegetables around the primary healthcare services in 2013 (*n* 272) and in 2018 (*n* 265).

**Results:**

Fruit diversity increased by 13·4 % (*P* < 0·001) and vegetables variety and quality by 16·1 % (*P* = 0·003) and 12·5 % (*P* < 0·001), respectively. Corn snacks showed an increase in availability (13·5 %; *P* = 0·002). The increase in advertising was observed for fruits and vegetables (34·6 %; *P* < 0·001) and ultra-processed foods (47·6 %; *P* < 0·001). Supermarkets showed an increase in the Healthy Food Store Index (three points; *P* < 0·001), while fruits and vegetables stores showed a decrease of one point in the index (*P* < 0·001).

**Conclusions:**

The unequal changes in the consumers’ food environment according to the food stores types demonstrate the importance of food supply policies that promote a healthy environment and favour the maintenance of traditional healthy food retailers.

The increase in the prevalence of obesity and other noncommunicable diseases is associated with dietary changes, such as increased consumption of ultra-processed foods (UPF) and decreased consumption of in natura foods, such as fruits and vegetables (FV) and culinary preparations^(1)^. Potential determinants of these changes include the community and the consumers’ food environments and their changes over time^(2)^.

The community environment is characterised by the distribution of food stores represented by the number, type, location and accessibility^(3)^. The literature has shown changes in different contexts. In the UK, changes in the food environment were observed between 1990 and 2008, particularly in the poorest areas^(4)^. In the USA, between 2009 and 2017, a significant decrease in the number of convenience stores was observed in low- and middle-income areas, while an increase in the number of supermarkets was observed in middle-income areas^(5)^. In Latin America, changes in the food environment indicate the growth of large supermarkets and convenience stores and the decline of traditional retail stores, such as local markets^(6)^.

Already, the consumer environment is represented by the availability, price, quality, promotion, variety and arrangement of foods in stores^(3)^. In cross-sectional studies, the consumers’ food environment characteristics that favour the consumption of UPF, such as increased availability, advertising and supply, have been associated with the high prevalence of chronic diseases^(6)^. Concurrently, a reduced availability of FV, low variety of vegetables and their poor quality have been associated with health risks^(6–9)^.

The consumers’ environment has been the target of several intervention studies that aim to influence consumers’ purchasing habits in ways that affect health and nutrition. Interventions in the arrangement and availability of food in the markets, advertising and the provision of nutritional information have shown potential for changing purchasing habits^(10)^. In this way, public policies aimed at the consumer environment, such as the government subsidies and taxes, are strategies used in different countries to increase the supply of healthy foods and reduce the consumption of unhealthy foods^(11)^. However, in some countries, such as Brazil, policies to promote a healthy food environment are stagnating. Some local governments propose supply policies that encourage open-air markets or stores specialised in FV. However, there are no policies focusing on aspects of the consumers’ food environment such as availability, variety, quality, price and advertising of food^(12,13)^.

Despite little regulatory intervention, changes in the food environment may occur as a result of changes in consumer behaviour, who may start to prefer healthier foods or choose to buy cheap foods in times of crisis^(10)^. In this sense, the WHO claims the importance of monitoring and evaluating the food environment and policies around the world^(2)^. However, there is a lack of knowledge about how the consumer food environment is changing in developing countries.

In this context, using data from stores’ audit, this study aimed to investigate the changes over a 5-year period in the consumers’ food environment in stores and open-air markets selling FV in the area of a health promotion service in Brazilian primary health care (PHC), called Health Academy Program (*Programa Academia da Saúde, PAS, in Portuguese*).

The PAS is a free health service that offers health promotion actions. Studies carried out in the PAS show that the interventions in the community have the potential to increase physical activity among the population in the area, regardless of participation in the service^(14–17)^. Proximity to the PAS was also associated with reports of greater ease of walking^(14)^. A possible explanation for these observations is related to the visual identity that encourages healthy habits in the general population. In addition, the presence of a health promotion service represents an important change in the physical environment of the neighbourhood and may support differences in healthy lifestyle habits^(15)^. Therefore, our hypothesis is that the consumers’ food environment in the area with PHC services has undergone changes that favoured health habits over time, in line with the potential for building a healthy environment in the territory. The possible influence of the health service on changes in the built environment is based on the particular standard of health that results from a system in which individuals interact with each other and with their environment and in which both individuals and environments adapt and change over time^(18)^.

## Materials and methods

### Setting and study design

The PAS units of Belo Horizonte, Minas Gerais, Brazil, were the setting for this longitudinal study with 5 years of follow-up. The city is the sixth most populous in the country^(19)^ and a national reference for effective PHC services throughout Brazil. Belo Horizonte was also one of the first cities to develop and implement PAS.

PAS has been a health promotion service of the PHC in Belo Horizonte since 2006 and a national service since 2011. PAS units are a space with infrastructure, equipment and human resources for health care. To achieve this, service users have access to free supervised physical activity, nutrition interventions, health promotion and health education, as well as complementary and integrative care^(20)^. Thus, PAS was used as the research setting for this study because it represents an important initiative to promote health and to prevent and control the transmission of noncommunicable diseases, mainly among people with high health vulnerability^(21)^.

### Study sample

The study sample was calculated taking into account the total number of PAS units in the city. The inclusion criteria of the units were they had to be open during the morning (as this is the predominant service operating shift) and be located in an area of medium and high vulnerability according to the Health Vulnerability Index^1^ (HVI)^(22)^ (priority areas for insertion in the service according to the municipal health policy); they could not have been the target of food and nutrition interventions in the last 24 months prior to the start of this study; and they had to be operating in 2012 (period of the sampling process)^(23)^.

Of the fifty units operating at the time of the sampling process, forty-two were eligible. Eighteen units were randomly selected for the study through simple random sampling, with two units per administrative district. The selection of PAS units for the study was done through an online draw based on a list of all eligible units with district and HVI information. If the second unit drawn from the district did not have an HVI similar to the first drawn, a new draw was carried out until similar HVI was obtained in order to obtain paired sociodemographic characteristics in the units. The steps of the sampling procedure are presented in the online supplementary material, Supplemental Material 1. These eighteen units were representative of PAS units with medium and high health vulnerability in Belo Horizonte, with a 95 % confidence level and <1·4 % error. More information on the methods and sampling can be found in Menezes *et al.* (2017)^(23)^.

To characterise the consumer food environment, this study examined food stores selling FV and open-air food markets located within a 1-mile (1600 m) buffer area around each PAS unit sampled. This radius was chosen because it includes 95 % of the adult destinations within a walkable distance^(24,25)^. In this study, only stores selling FV were evaluated, to deepen studies on these food groups regarding their power in promoting health^(26)^.

### Data collection

The data collection was performed through an audit of eligible stores and open-air food markets in two periods: the baseline in 2013 and after 5 years in 2018. The research team consisted of dieticians or undergraduate students of nutrition. They performed the audit in pairs, supervised by a researcher. All interviewers were trained using a field manual proposed by the ‘Obesogenic Environment Study (ESAO)’ in Sao Paulo, Brazil (2010)^(27)^, which translated and validated the tool for assessing the consumer food environment, called ESAO-S, used in this study. Data consistency was performed by the field supervisor who produced weekly reports. After entering the data, descriptive analyses of all variables were performed in order to identify unusual and missing values, and if necessary, the questionnaires were returned for correction^(28)^.

The City Hall provided a complete list of food stores registered in the municipality, which contained the following information: brand name, record number at the National Register of Legal Entities and address. The address of each open-air market was obtained from the website of the Municipal Supply Department. At baseline, the eligible stores and open-air food markets were identified from these lists. During the audit, data on the commercial name, National Register of Legal Entities and address of the store were confirmed. The name and address of the stores and open-air food markets contained in the buffer area were selected to guide the interviewers in the audit. If the store in the public database was not found during the audit, contact by telephone or search using Google Street View search was used. Non-registered stores that were identified during the audit conducted in the buffer area were also included, allowing the database to be updated^(28)^.

In 2018, a new audit was conducted. The audit used the list of names and addresses of stores and open-air food markets with data collected in 2013. New stores randomly found in the buffer area during this audit were also included. More information can be seen in Costa (2018)^(28)^.

To ensure the quality of the data, training sessions were also held every 6 months in 2018, and all other precautions taken in 2013 to ensure the quality of the data adopted were again applied^(29)^.

### Consumer food environment

In both periods, information on consumers’ food environment was assessed using ESAO-S^(29)^. This tool was constructed by adapting several existing instruments: the Nutrition Environment Measures Survey in Stores^(30)^, the Environmental Profile of a Community’s Health^(31)^, the Nutrition Environment Measures Survey in Restaurant^(32)^ and the in-store measurement tool developed by Ball *et al.* ^(33)^. In addition, they realised a panel of experts in food environment from Australia, Europe and the USA^(29)^.

The ESAO-S evaluates fresh FV and UPF. For fresh FV, the following were assessed: diversity, variety, quality, advertising, section near the main entrance and price; and for UPF: availability, presence in the FV section, variety, advertising and price. The presence of UPF in the FV section and the variety of UPF were not evaluated in the open-air markets^(29)^.

The ESAO-S was adapted for the ten most frequently purchased fruits (banana, orange, papaya, watermelon, apple, mango, pineapple, tangerine, grape, melon and pumpkin) and vegetables (chayote, tomato, carrot, lettuce, zucchini, cabbage, beetroot, kale and okra) in Belo Horizonte^(34)^. Tubers and roots were not considered in the FV assessments, considering that they are foods rich in carbohydrates. The evaluated UPF included the five most consumed products (regular soda, fruit-flavoured drinks and juice/nectars with added sugar, cream-filled chocolate cookies and corn chip snacks) in Brazil^(34)^. For open-air markets, sweetened beverages were only evaluated together and in food stores separately (regular soda or fruit-flavoured drinks or juice/nectars with added sugar).

The availability of FV and UPF was assessed by the presence of at least one item (yes/no). The diversity of FV was defined by the number of FV among the ten items examined (number). The variety of FV was determined by the number of different types of items (e.g. iceberg lettuce, green-leaf lettuce, red-leaf lettuce) and of UPF by different brands and flavours (number). For the analysis, *the values of the discrete variables were categorised according* to score points of the *Healthy Food Store Index* (HFSI) (see below and in the online supplementary material, Supplemental Material 2). For fruit diversity, the categories were (0) if not available; (1) 1–7 types of the ten most purchased fruits are available; and (2) 8–10 of the ten most purchased fruits are available. The variety categories were (0) if not even one fruit variety is available; (1) if up to fourteen varieties are available; and (2) if fifteen or more varieties are available. For vegetables, the diversity categories were (0) if not available; (1) if 1–7 types of the ten most purchased vegetables are available; and (2) 8–10 of the ten most purchased vegetables are available and variety: (0) if not even one vegetable variety is available; (1) if up to fourteen varieties are available; and (2) if fifteen or more varieties are available.

The quality of FV was assessed by determining whether most of the food was wilted, bruised, overripe or looked old (good/bad)^(35)^.

The FV and UPF advertising was available when it had a printed material containing messages or images, tasting counters, demonstration or distribution samples, pennants, posters and banners, displays or folders (presence/absence)^(35)^.

The price index of FV and UPF was analysed using the *z*-score scale. This makes it possible to compare the prices of the different items surveyed. The *z*-score scale has a mean of zero and a sd of one and indicates how many sds the variable is from the mean^(35)^. It is obtained by subtracting the mean price (of each food item throughout all the food stores) from each observation and then dividing the difference by the sd.

### Healthy food store index

The HFSI was used to evaluate the access to healthy food. The index varies from 1 to 16 and consists of the availability, variety and advertising of healthy items (FV) and UPF (sweetened beverages, corn chips and cream-filled chocolate cookies)^(36)^. The scoring system for the HFSI is included in the online supplementary material, Supplemental Material 2. The higher HFSI score indicates better access to healthy foods and, consequently, lower access to UPF in the area^(29)^.

### Type of stores

The stores were classified into categories adapted from a Brazilian study^(29)^: (i) supermarkets; (ii) FV markets (specialised stores or open-air food markets); and (iii) local markets (local grocery stores, convenience stores, delis and bakeries).

### Data analysis

The consumers’ food environment was described for the period of 2013–2018. The absolute number, frequency, 95 % confidence intervals, median and interquartile range (P_25_–P_75_) have been described by year and type of store.

The percentage of change in the consumer food environment was calculated by the difference between the information observed in 2018 and 2013 (change = 2018 measure – 2013 measure). Negative values indicate a decrease over time, while positive values indicate an increase over time. The change analyses were also stratified by store type. To estimate the CI, a proportion test with database time was used.

To assess the change over time, a database was created containing information from all stores and open-air food markets collected in both periods and a variable indicating time. For categorical variables, the *χ* ^2^ test was used. The two-sample test of proportions using groups was used to estimate the CI of the difference in proportions. For continuous variables, the Mann–Whitney *U* test was used due to asymmetric distribution. Data were analysed using Stata version 14, and *P* < 0·05 was considered significant.

This study does not involve human data. However, those responsible for the commercial establishments authorised the audit and signed a written informed consent form. The protocol was approved by the Ethics Committee University and the City Hall.

## Results

**Fig. 1:**
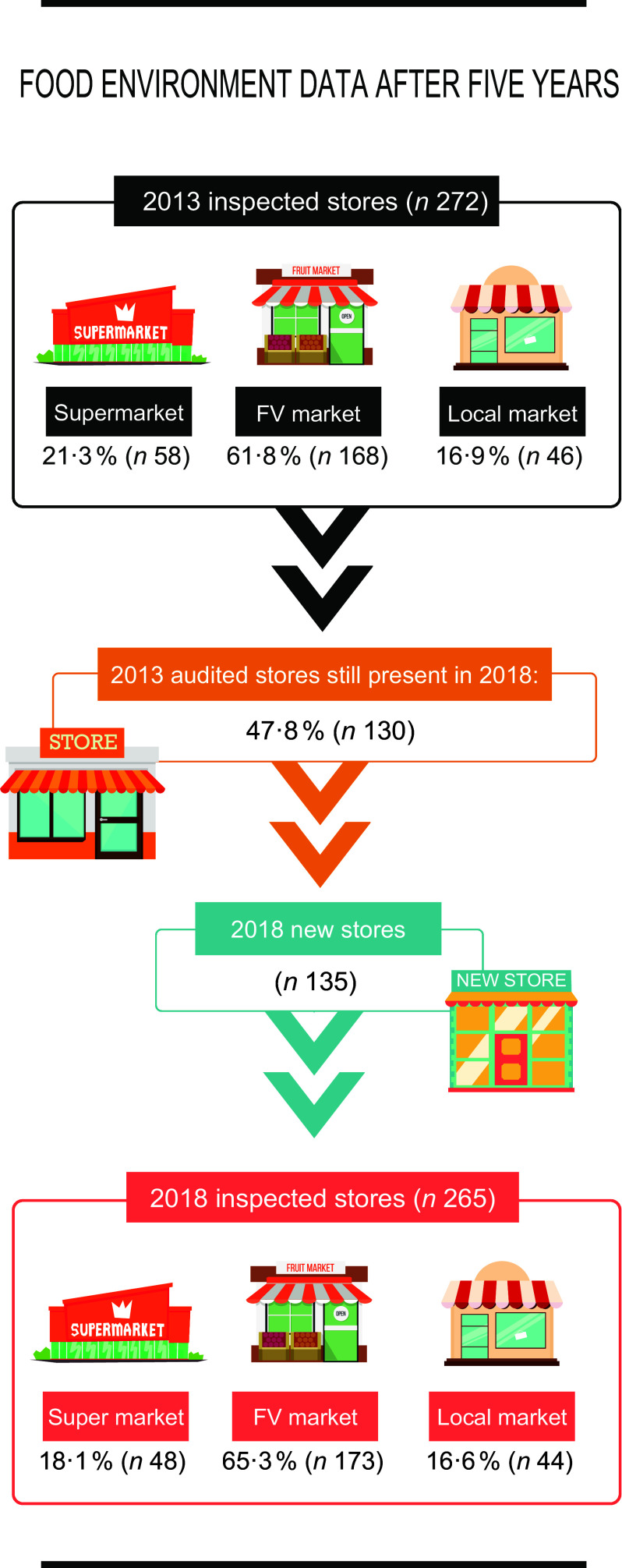
Food environment data after 5 years. Belo Horizonte, Minas Gerais, Brazil, 2013–2018. FV, fruits and vegetables

In 2013, 272 stores and open-air food markets were audited. After 5 years, 47·8 % of these stores were found, and 135 new stores were identified, totalising 265 stores and open-air food markets audited in 2018. In both of the evaluated periods, the FV market was the most predominant type of store (2013: 61·8 %; 2018: 65·3 %), followed by supermarkets (2013: 21·3 %; 2018: 18·1 %) and local markets (2013:16·9 % 2018: 16·6 %) (Fig. 1).

After 5 years, the evaluation of the consumers’ food environment revealed a growth of 13·4 % in fruit diversity (8–10 types) and 16·1 % in vegetable variety (fifteen or more types). An increase in the number of stores and open-air food markets, with a higher quality of vegetables, was also observed (12·5 %) (Table 1).

**Table 1:** Characteristics of the consumer food environment. Belo Horizonte, Minas Gerais, Brazil, 2013–2018

Characteristics of the consumer food environment	2013 (*n* 272)			2018 (*n* 265)			Difference		*P* value
	n	% (95 % CI) or median (IQR)		n	% (95 % CI) or median (IQR)		Points % (95 % CI) or difference of median		
FV section near the main entrance†	216	79·4	74·1, 83·8	199	78·0	72·5, 82·7	–1·4	–5·6, 8·3	0·700‡
Fruit diversity									<0·001*,‡
* 0*	4	1·5	0·0, 0·4	9	3·4	1·2, 6·4	1·9	–4·5, 6·8	
* 1–7*	95	34·9	29·4, 40·8	52	19·6	15·2, 24·9	–15·3	–22·7, –7·8	
* 8–10*	173	63·6	57·7, 69·1	204	77	71·5, 81·7	13·4	5·7, 21·0	
Vegetables diversity									0·924‡
* 0*	7	2·6	1·2, 5·3	8	3	1·5, 5·9	0·4	–0·2, 3·2	
* 1–7*	37	13·6	10·0, 18·2	34	12·8	9·2, 17·4	–0·8	–6·5, 4·9	
* 8–10*	228	83·8	78·9, 87·8	223	84·1	79·2, 88·9	0·3	–5·8, 6·5	
Fruit variety									0·167‡
* 0*	4	1·5	0·0, 3·9	9	3·4	1·8, 6·4	1·9	–4·5, 0·6	
* 1–14*	108	39·7	34·0, 45·7	90	34	28·4, 39·9	–5·7	–13·9, 2·4	
* 15 or more*	160	58·8	52·8, 64·5	166	62·6	56·6, 68·3	3·8	–4·4, 12·0	
Vegetables variety									<0·001*,‡
* 0*	7	2·6	1·2, 5·3	8	3	1·5, 5·9	0·4	–2·3, 3·2	
* 1–14*	203	74·6	69·1, 79·5	154	58·1	52·0, 63·9	–16·5	–24·4, –8·6	
* 15 or more*	62	22·8	18·2, 28·2	103	38·9	33·1, 44·9	16·1	8·3, 23·7	
Quality of fruit									0·295‡
* 75 % were evaluated as good*	163	78·0	71·8, 83·1	208	81·9	76·6, 86·2	3·9	–3·4, 11·2	
* 25 % were evaluated as bad*	46	22·0	16·9, 28·1	46	18·1	13·8, 23·4	–3·9	–11·2, 3·4	
Quality of vegetables									0·003*,‡
* 75 % were evaluated as good*	148	62·7	56·3, 68·7	191	75·2	69·5, 80·1	12·5	4·3, 20·6	
* 25 % were evaluated as bad*	88	37·3	31·3, 43·7	63	24·8	19·8, 30·5	–12·5	–20·6, –4·0	
Presence of UPF in FV section†	172	66·1	60·0, 71·7	118	46·3	40·2, 52·5	–19·8	–28·3, –11·0	<0·001*,‡
UPF availability									
* Soda* †	142	54·6	48·5, 60·6	130	49·1	43·9, 56·9	–5·5	–14·1, 2·9	0·203§,‡
* Fruit-flavoured drinks and juice/nectars* †	134	51·5	45·4, 57·6	133	52·2	46·0, 58·3	0·7	–0·8, 9·2	0·888§,‡
* Sweetened drinks (soda, fruit-flavoured drinks and juice/nectars)*	134	51·5	44·7, 56·8	143	56·1	49·9, 62·1	4·6	12·3, 4·9	0·701§,‡
* Cream-filled chocolate cookies*	111	40·8	35·1, 46·8	95	40·9	34·7, 47·4	0·1	–8·4, 8·7	0·975§,‡
* Corn chips snacks*	105	38·6	33·0, 44·6	199	44·9	38·9, 50·9	6·3	–2·0, 14·6	0·139§,‡
UPF variety†,‖									
* Soda*	139	13·0	9·0, 25·0	129	18·0	9·0, 24·0	3·0	–	0·336§
* Fruit-flavoured drinks and juice/nectars*	132	6·0	3·0, 14·0	132	8·0	3·5, 16·0	2·0	–	0·452§
* Sweetened drinks (soda, fruit-flavoured drinks and juice/nectars)*	147	21·0	9·0, 37·0	142	27·0	9·0, 41·0	6·0	–	0·405§
* Cream-filled chocolate cookies*	109	5·0	3·0, 8·0	92	4·0	2·0, 6·5	–1·0	–	0·020*,§
* Corn chips snacks*	95	1·0	1·0, 2·0	97	4·0	2·0, 4·0	3·0	–	<0·001*,§
Advertising†									
* FV*	63	24·2	19·3, 29·8	255	58·8	52·6, 64·7	34·6	26·6, 42·7	<0·001*,‡
* UPF*	68	26·1	21·1, 31·9	188	73·7	67·9, 78·8	47·6	39·9, 55·1	<0·001*,‡
Price index‖									
* FV*	274	–0·19	–0·53, 0·17	261	–0·12	–0·37, 0·24	0·07	–	0·298§
* UPF* †	76	–0·11	–0·53, 0·36	141	0·05	–0·33, 1·0	0·16	–	0·003*,§

FV, fruit and vegetable; UPF, ultra-processed food. The increase or decrease in values of change does not mean improvement or worsening, respectively. The indication of improvement or worsening will depend on the analysed variable; common sense in interpretation is important.

* *P* > 0.05.

† Not investigated at open-air food markets.

‡ *χ* ^2^.

§ Mann–Whitney.

‖ Median(P25; P75).

The presence of UPF in the FV section decreased from 66·1 % to 46·3 % (Δ = –19·8 %; 95 % CI: –28·3, –11·0). The variety of cream-filled chocolate cookies had also decreased by 2 % (Table 1). However, there was an increase in the variety of corn chip snacks (Δ = 13·5 % and Δ = 3·0 %, respectively).

Food advertising showed strong growth during the period. UPF advertising increased from 26·1 % to 73·7 % in 5 years and of FV from 24·2 % to 34·6 %. On the other hand, the price index varied only for the UPF, indicating an increase in its costs (Table 1).

The analysis by type of store showed an increase in the diversity of fruits in supermarket (Δ = 18·9 %; 95 % CI: 4·3;33·4), while in the FV market and in the local market, there was a decrease in stores offering between one and seven types of fruit (Δ = –11·8 %; 95 % CI: –20·4, –3·1 and Δ = –24·0; 95 % CI: –43·9, –4·9, respectively). An increase in the variety of vegetables was only observed in supermarkets (Δ = 40·4 %; 95 % CI: 23·7, 57·0), and the improvement in the quality of vegetables (Δ = 13·1 %; 95 % CI: 3·4, 22·8) and the decrease in the presence of UPF in the FV section were both observed only in FV markets (Δ = –27·7 %; 95 % CI: –38·2, –17·2) (Table 2a–c).

**Table 2a:** Characteristics of the food environment by type of stores. Belo Horizonte, Minas Gerais, Brazil, 2013–2018

Characteristics of the consumer food environment	Supermarket								
	2013 (*n* 58)			2018 (*n* 48)			Difference		*P* value
	n	% (95 % CI) or median (IQR)		n	% (95 % CI) or median (IQR)		Points % (95 % CI) or difference of median		
FV section near the main entrance†	26	44·8	32·2, 58·1	22	45·8	31·9, 60·4	1·0	–18·0, 20·0	0·918‡
Fruit diversity									0·017*,‡
* 0*	0	0		0	0		0	–	
* 1–7*	17	29·3	18·8, 42·6	5	10·4	4·2, 23·3	–18·9	–33·4, –4·3	
* 8–10*	41	70·7	57·4, 81·2	43	89·6	76·7, 95·7	18·9	4·3, 33·4	
Vegetables diversity									0·056‡
* 0*	0	0		0	0		0	–	
* 1–7*	9	15·5	8·1, 27·6	2	4·2	0·9, 15·9	–11·3	–22·2, 0·0	
* 8–10*	49	84·5	72·3, 91·9	46	95·8	84·1, 99·0	11·3	22·0, 0·0	
Fruit variety									0·237‡
* 0*	0	0		0	0		0	–	
* 1–14*	22	37·9	26·1, 51·3	13	27·1	16·1, 41·8	–10·8	–28·6, 6·9	
* 15 or more*	36	62·1	48·6, 73·9	35	72·9	58·2, 83·9	10·8	–6·9, 28·6	
Vegetables variety									<0·001*,‡
* 0*	0	0		0	0		0	–	
* 1–14*	50	86·2	74·3, 93·1	22	45·8	31·9, 60·4	–40·4	–57·0, –23·7	
* 15 or more*	8	13·8	6·9, 25·7	26	54·2	39·6, 68·0	40·4	23·7, 57·0	
Quality of fruit									0·131‡
* 75 % were evaluated as good*	39	90·7	76·9, 96·6	47	97·9	85·8, 99·7	7·2	–2·3, 16·8	
* 25 % were evaluated as bad*	4	9·3	3·3, 23·0	1	2·1	0·1, 14·2	–7·2	–16·8, 2·3	
Quality of vegetables							0·0		0·213‡
* 75 % were evaluated as good*	33	63·5	49·2, 75·7	36	75·0	60·3, 85·5	11·5	–6·4, 29·5	
* 25 % were evaluated as bad*	19	36·5	24·3, 50·8	12	25·0	14·4, 39·6	–11·5	–29·5, 6·4	
Presence of UPF in FV section†	45	77·6	64·7, 86·7	39	81·2	67·3, 90·2	3·6	–11·7, 19·0	0·643‡
UPF availability									
* Soda* †	56	96·5	86·7, 99·2	48	100·0	–	3·5	–1·2, 8·1	0·194‡
* Fruit-flavoured drinks and juice/nectars* †	56	96·5	86·7, 99·2	48	100·0	–	3·5	–1·2, 8·1	0·194‡
* Sweetened drinks (soda, fruit-flavoured drinks and juice/nectars)*	56	96·5	86·7, 99·2	48	100·0	–	3·5	–1·2, 8·1	0·194‡
* Cream-filled chocolate cookies*	56	96·5	86·7, 99·2	41	97·6	83·8, 99·7	1·1	–5·5, 7·6	0·757‡
* Corn chips snacks*	54	93·1	82·5, 97·5	48	100·0	–	6·9	–13·4, 0·0	0·064‡
UPF variety†,‖									
* Soda*	56	25·5	19·5, 31·0	48	24·0	22·0, 27·0	–1·5	–	0·345§
* Fruit-flavoured drinks and juice/nectars*	56	14·5	9·5, 17·0	48	17·0	15·0, 19·5	2·5	–	0·012*,§
* Sweetened drinks (soda, fruit-flavoured drinks and juice/nectars)*	56	38·5	32·0, 46·5	48	42·0	35·0, 45·0	3·5	–	0·215§
* Cream-filled chocolate cookies*	56	7·0	6·0, 8·0	41	6·0	5·0, 9·0	–1·0	–	0·228§
* Corn chips snacks*	53	2·0	1·0, 2·0	48	4·0	3·0, 4·0	2·0	–	<0·001*,§
Advertising†									
* FV*	17	29·3	18·8, 42·6	31	64·6	49·7, 77·1	35·3	17·4, 53·2	<0·001*,‡
* UPF*	23	60·3	46·9, 72·3	4	8·33	3·0, 20·8	–52·0	–66·8, –37·2	<0·001*,‡
Price index‖									
* FV*	56	–0·30	–0·58, –0·06	48	–0·1	–0·31, 0·13	0·2	–	0·303§
* UPF* †	51	–0·16	–0·51, 0·29	48	–0·39	–0·66, –0·21	–0·2	–	0·168§

FV, fruit and vegetable; UPF, ultra-processed food. The increase or decrease in values of change does not mean improvement or worsening, respectively. The indication of improvement or worsening will depend on the analysed variable; common sense in interpretation is important.

* *P* > 0.05.

† Not investigated at open-air food markets.

‡ *χ* ^2^.

§ Mann–Whitney.

‖ Median(P25; P75).

**Table 2b:** Characteristics of the food environment by type of stores. Belo Horizonte, Minas Gerais, Brazil, 2013–2018

Characteristics of the consumer food environment	Fruit and vegetables market								
	2013 (*n* 168)			2018 (*n* 163)			Difference		*P* value
	n	% (95 % CI) or median (IQR)		n	% (95 % CI) or median (IQR)		Points % (95 % CI) or difference of median		
FV section near the main entrance†	167	99·4	95·8, 99·9	158	96·9	92·8, 98·7	–2·5	–5·3, 0·4	0·092‡
Fruit diversity									
* 0*	4	2·3	0·1, 6·2	7	4·0	1·9, 8·3	1·7	–2·0, 5·4	0·025*,‡
* 1–7*	46	27·4	21·1, 34·7	27	15·6	10·9, 21·9	–11·8	–20·4, –3·1	
* 8–10*	118	70·2	62·8, 76·7	139	80·3	73·7, 85·6	10·1	1·0, 19·2	
Vegetables diversity									0·602‡
* 0*	6	3·5	1·5, 7·8	6	3·5	1·5, 7·6	0	–4·0, 0·4	
* 1–7*	9	5·3	2·8, 10·0	14	8·1	4·8, 13·3	2·8	–2·5, 8·0	
* 8–10*	153	91·1	85·6, 94·6	153	88·4	82·7, 92·4	–2·7	–9·0, 3·7	
Fruit variety									0·652‡
* 0*	4	2·3	0·1, 6·2	7	4·0	1·9, 8·3	1·7	–0·2, 5·4	
* 1–14*	50	29·8	23·3, 37·2	48	27·7	21·5, 34·9	–2·1	–11·6, 7·5	
* 15 or more*	114	67·8	50·3, 74·5	118	68·2	60·8, 74·8	0·4	–9·5, 10·2	
Vegetables variety									0·071‡
* 0*	6	3·5	1·5, 7·8	6	3·5	1·5, 7·6	0	–4·0, 3·8	
* 1–14*	112	66·7	59·1, 73·4	95	54·9	47·3, 62·2	–11·8	–22·0, –1·4	
* 15 or more*	50	29·8	23·2, 37·2	72	41·6	34·4, 49·2	11·8	1·7, 21·0	
Quality of fruit									0·512‡
* 75 % were evaluated as good*	110	76·9	69·2, 83·1	132	80·0	73·1, 85·5	3·1	–6·1, 12·2	
* 25 % were evaluated as bad*	33	23·1	16·8, 30·8	33	20·0	14·5, 26·9	–3·1	–12·2, 6·1	
Quality of vegetables									0·008*,‡
* 75 % were evaluated as good*	101	66·9	58·9, 74·0	132	80·0	73·1, 85·5	13·1	3·4, 22·8	
* 25 % were evaluated as bad*	50	33·1	26·0, 41·1	33	20·0	14·5, 26·9	–13·1	–22·8, –3·4	
Presence of UPF in FV section†	93	59·6	51·6, 67·1	52	31·9	25·1, 39·5	–27·7	–38·2, –17·2	<0·001*,‡
UPF availability									
* Soda* †	53	31·4	24·5, 39·2	38	21·9	16·4, 28·8	–9·5	–18·9, –0·0	0·052‡
* Fruit-flavoured drinks and juice/nectars* †	39	25·0	18·8, 32·5	44	27·0	20·6, 34·4	1·9	–7·6, 11·6	0·685‡
* Sweetened drinks (soda, fruit-flavoured drinks and juice/nectars)*	53	31·5	24·9, 39·0	51	31·3	24·5, 38·9	–0·2	–10·2, 9·7	0·959‡
* Cream-filled chocolate cookies*	20	11·9	7·8, 17·8	20	13·2	8·6, 19·6	1·3	–6·0, 8·5	0·735‡
* Corn chips snacks*	17	10·12	6·3, 15·7	29	16·8	11·8, 23·1	6·6	–0·5, 13·8	0·072‡
UPF variety†,‖									
* Soda*	47	8·0	5·0, 11·0	37	6·0	5·0, 10·0	–2·0	–	0·352§
* Fruit-flavoured drinks and juice/nectars*	39	2·0	1·0, 4·0	43	2·0	1·0, 4·0	0	–	0·535§
* Sweetened drinks (soda, fruit-flavoured drinks and juice/nectars)*	53	8·0	4·0, 13·0	50	6·0	1·0, 12·0	–2·0	–	0·161§
* Cream-filled chocolate cookies*	18	2·0	1·0, 3·0	17	1·0	1·0, 2·0	–1·0	–	0·065§
* Corn chips snacks*	11	1·0	1·0, 1·0	16	2·5	1·0, 4·0	1·5	–	0·009*,§
Advertising†									
* FV*	42	26·9	20·5, 34·5	103	63·2	55·4, 70·3	36·3	26·1, 46·4	<0·001*,‡
* UPF*	16	10·2	6·3, 16·2	154	94·5	89·7, 97·1	84·3	78·0, 90·1	<0·001*,‡
Price index‖									
* FV*	150	–0·10	–0·55, 0·39	171	–0·14	–0·37, 0·31	–0·04	–	0·586§
* UPF* †	6	0·27	0·22, 0·57	50	1·34	0·52, 1·78	1·07	–	0·085§

FV, fruit and vegetable; UPF, ultra-processed food. The increase or decrease in values of change does not mean improvement or worsening, respectively. The indication of improvement or worsening will depend on the analysed variable; common sense in interpretation is important.

* *P* > 0.05.

† Not investigated at open-air food markets.

‡ *χ* ^2^.

§ Mann–Whitney.

‖ Median(P25; P75).

**Table 2c:** Characteristics of the food environment by type of stores. Belo Horizonte, Minas Gerais, Brazil, 2013–2018

Characteristics of the consumer food environment	Local market								
	2013 (*n* 46)			2018 (*n* 44)			Difference		*P* value
	n	% (95 % CI) or median (IQR)		n	% (95 % CI) or median (IQR)		Points % (95 % CI) or difference of median		
FV section near the main entrance†	23	50·0	35·4, 64·6	19	43·2	29·0, 58·5	–6·8	–27·3, 13·7	0·517‡
Fruit diversity									0·039*,‡
* 0*	0	0	–	2	4·5	1·2, 17·2	4·5	–1·6, 10·7	
* 1–7*	32	69·5	54·3, 81·4	20	45·5	31·0, 60·7	–24·0	–43·9, –4·2	
* 8–10*	14	30·4	18·5, 45·6	22	50·0	35·1, 64·9	19·6	0·0, 39·4	
Vegetables diversity									0·820‡
* 0*	1	2·1	0·1, 14·8	2	4·5	1·2, 17·2	2·4	–5·0, 9·8	
* 1–7*	19	41·3	27·7, 56·4	18	40·9	27·0, 56·4	–0·4	–20·7, 19·9	
* 8–10*	26	56·5	41·5, 70·4	24	54·5	39·2, 69·0	–2	–22·5, 18·5	
Fruit variety									0·212‡
* 0*	0	0		2	4·5	1·1, 17·2	4·5	–1·6, 10·7	
* 1–14*	36	78·2	63·5, 88·2	29	65·9	50·2, 78·7	–12·3	–30·7, 6·0	
* 15 or more*	10	21·7	11·8, 36·5	13	29·5	39·3, 69·0	7·8	–10·2, 25·8	
Vegetables variety									0·739‡
* 0*	1	2·1	0·1, 14·8	2	4·5	1·1, 17·2	2·4	–5·1, 9·8	
* 1–14*	41	89·1	75·7, 95·5	37	84·1	69·5, 92·4	–5·0	–19·1, 9·0	
* 15 or more*	4	8·7	0·3, 21·6	5	11·4	4·6, 25·2	2·7	–9·7, 15·1	
Quality of fruit									0·420‡
* 75 % were evaluated as good*	9	39·1	20·6, 61·4	12	29·3	17·0, 45·5	31·6	11·6, 50·8	
* 25 % were evaluated as bad*	14	60·9	38·6, 79·4	29	70·7	54·5, 83·0	–31·6	–36·4, 9·0	
Quality of vegetables									0·242‡
* 75 % were evaluated as good*	14	42·4	26·3, 60·4	23	56·1	40·1, 70·9	13·7	–9·0, 36·4	
* 25 % were evaluated as bad*	19	57·5	39·5, 73·8	18	43·9	29·1, 59·8	–13·6	–36·4, 9·0	
Presence of UPF in FV section†	34	73·9	58·8, 84·9	27	61·4	45·8, 74·9	–12·5	–31·7, 6·7	0·203‡
UPF availability									
* Soda* †	37	80·4	65·8, 89·7	44	100·0	–	19·6	8·1, 31·0	0·002*,‡
* Fruit-flavoured drinks and juice/nectars* †	37	84·7	65·8, 89·7	41	93·2	80·1, 97·9	8·3	–4·3, 21·1	0·205‡
* Sweetened drinks (soda, fruit-flavoured drinks and juice/nectars)*	29	84·8	70·7, 92·8	44	100·0	–	15·2	4·8, 25·6	0·007*,‡
* Cream-filled chocolate cookies*	35	76·1	61·1, 86·5	34	89·5	74·2, 96·2	13·4	–2·3, 29·1	0·111‡
* Corn chips snacks*	34	73·9	58·8, 84·9	42	95·4	82·7, 98·2	21·5	7·4, 35·6	<0·001*,‡
UPF variety†,‖									
* Soda*	36	13·5	10·0, 19·0	44	18·5	13·5, 22·0	5·0	–	0·013*,§
* Fruit-flavoured drinks and juice/nectars*	37	5·0	4·0, 9·0	41	9·0	8·0, 12·0	4·0	–	0·021*,§
* Sweetened drinks (soda, fruit-flavoured drinks and juice/nectars)*	38	19·0	13·0, 27·0	44	27·5	20·0, 33·0	8·5	–	0·009*,§
* Cream-filled chocolate cookies*	35	4·0	3·0, 7·0	34	3·0	2·0, 5·0	–1·0	–	0·378§
* Corn chips snacks*	31	1·0	1·0, 2·0	33	3·0	2·0, 4·0	2·0	–	<0·001*,§
Advertising†									
* FV*	4	8·7	3·2, 21·6	16	36·4	23·2, 51·9	27·7	11·3, 44·0	0·002*,‡
* UPF*	17	36·9	23·0, 50·9	30	68·2	52·5, 80·6	31·2	11·6, 50·8	0·003*,‡
Price index‖									
* FV*	41	0·07	–0·28, 0·12	42	–0·13	–0·39, 0·21	–0·2	–	0·610§
* UPF* †	19	0·21	–0·15, 5·6	43	0·05	–0·2, 0·58	–0·16	–	0·957§

FV, fruit and vegetable; UPF, ultra-processed food. The increase or decrease in values of change does not mean improvement or worsening, respectively. The indication of improvement or worsening will depend on the analysed variable; common sense in interpretation is important.

* *P* > 0.05.

† Not investigated at open-air food markets.

‡ *χ* ^2^.

§ Mann–Whitney.

‖ Median(P25; P75).

In terms of UPF availability, the availability of corn chip snacks, soda and sweetened beverages increased in local markets (Δ = 15·2 %; 95 % CI: 7·4, 35·6, Δ = 19·6 %; 95 % CI: 8·1, 31·0 and Δ = 15·2 %; 95 % CI:4·8;25·6, respectively) (Table 2a–c). The variety of corn chip snacks increased in all types of stores (supermarket Δ = 2·0 median difference; FV market Δ = 1·5 median difference and local market Δ = 2·0 median difference). Similarly, soda, fruit-flavoured or nectar/juice and sweetened drinks variety also increased in local market (Δ = 5·0 median difference, Δ = 4·0 median difference and 8·5 median difference, respectively) (Table 2a–c). In supermarkets, the fruit-flavoured or nectar/juice variety also increased (Δ = 2·5 median difference).

The change in food advertising also varied by type of store. An increase in both types of advertising was observed in FV markets (FV advertisements: Δ = 36·3 % 95 % CI: 26·1, 46·4; UPF advertisements: Δ = 84·3 % 95 % CI: 78·0, 90·1) and in local markets (FV advertisements: Δ = 27·7 % 95 % CI: 11·3, 44·0; UPF advertisements: Δ = 31·2 % 95 % CI: 11·6, 50·8). In supermarkets, FV advertising increased (Δ = 35·3 %, CI95 %: 17·4, 53·2) and UPF advertising decreased (Δ = –52·0 %; –66·8, –37·2). No change in the price index was observed when analysed by type of store (Table 2a–c).

**Fig. 2:**
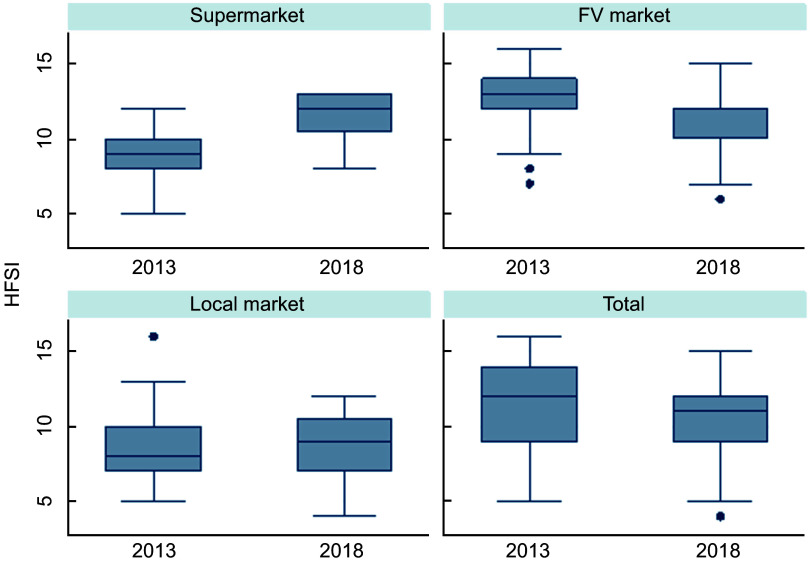
Median of healthy food store index (HFSI), according to the type of store. Belo Horizonte, Minas Gerais, Brazil, 2013. FV, fruits and vegetables. Mann–Whitney *U* test – total (*P* = 0·006); supermarket (*P* < 0·001); FV market (*P* < 0·001); local market (*P* = 0·357)

HFSI decreased over the 5 years analysed in this study (2013 = 12 (9–14) and 2018 = 11 (9–14)), but this result varied according to the type of store. Access to healthy foods improves in supermarkets (2013 = 9 (8–10) and 2018 = 12 (10·5–13); Δ = 3 points in HFSI); in contrast, in FV markets, it gets worse (2013 = 13 (12–14) and 2018 = 12 (10–12); Δ = –1 point in HFSI) (Fig. 2).

## Discussion

The results indicate that there have been changes in the consumers’ food environment that are favourable to the promotion of healthy diets, such as an improvement in fruit diversity and vegetable variety, vegetable quality, a reduction in the presence of UPF in FV sections, an increase in FV advertising and an increase in the UPF price index. However, an increase in the variety of corn chip snacks and an increase in UPF advertising have been also observed, which represents a barrier to healthy diets. In addition, in the set of stores and open-air food markets, there was a decrease in access to healthy foods (HFSI) in FV markets and an increase in supermarkets.

After 5 years, an improvement in the diversity, variety and quality of FV was observed. Characteristics of retail food are highlighted by consumers as the main factors that influence FV consumption^(37)^. So, some aspects of the consumers’ food environment can be used as a stimulus for healthy habits, such as promotions, advertisements and the arrangement of items in the store^(7,10,18)^. However, there is still room for improvement in the variety of FV, especially in local and FV markets, as the low variety of these foods has contributed to the monotony of FV consumption^(38)^ which may cause a loss of food culture. In addition, reducing the presence of UPF in the FV section, as observed in this study, is important to reduce competition for the purchase of FV.

A negative change in the consumers’ food environment identified in this study was an increase in the availability and variety of UPF. This means that there may be increased exposure of the population to UPF and negatively impact health conditions^(1,6,9)^. It is important to emphasise that this study analysed only stores selling FV, excluding other types of stores selling exclusively UPF, such as fast-food restaurants. In this sense, the access to UPF in the area may still be underestimated.

The changes in the consumers’ food environment varied according to the type of store. Beneficial changes for FV were found in supermarkets and FV market. The local markets were the stores with the highest increase in the availability and variety of UPF. This result is worrying as local markets are considered to be important outlets for the purchase of food for home consumption, especially in low-income areas^(6)^. In addition, the strengthening of local trade and small food stores, such as FV markets and local markets, is important in building a healthier and more sustainable food environment^(2)^.

In the present study, a general increase in FV and UPF advertising was observed, but in supermarkets, UPF advertising decreased. This situation can lead to strong competition with healthy foods in these stores^(39)^. One hypothesis for the increase in advertising is pressure from the food industry to display advertising in stores in exchange for commercial advantages with retailers^(39)^. In addition, the intensification of UPF advertising in the FV market may be a strategy to increase sales of products that are more profitable for the retailer, given the wide price variations that FV can suffer^(40)^. These hypotheses need to be tested in further studies.

Food advertising is recognised as an important obstacle to healthy diets because unhealthy foods advertising is more often than healthy foods ones^(41)^. Some studies have shown changes in food advertising, especially on television. In Australia, there was an 11 % reduction in the rate of food and beverage advertising between 2006 and 2008^(42)^. In Canada, on the other hand, there was a 4 % increase in the total number of food and beverage advertisements on television between 2011 and 2016, with sugary drinks and fast food being the most dominant^(43)^. There is still a lack of studies investigating changes in advertising in food stores^(10)^.

Public health advocates and international health agencies, including the WHO, are calling on governments to implement policies that restrict the advertising of unhealthy foods^(44)^. Examples of such policies include the Code of Broadcast Advertising in the UK^(45)^, the Special Act on Safety Management of Children’s Dietary Life Safety Management in South Korea^(46)^, the Consumer Protection Act in Canada^(47)^ and Chile’s Food Labeling and Advertising Law^(48)^. Evaluations of these policies found few or no policy-related reductions in unhealthy food advertising; however, not all policies have been evaluated^(44)^.

In Brazil, the rules related to food advertising are considered insufficient, and regulatory projects have been pending in Congress for more than 20 years. The regulation of the food environment is a subject of dispute and involves two central elements: political inertia and commercial conflicts of interests of the regulated sector, which affect the public regulatory agenda^(2)^. Concomitant to the high publicity, Brazilian consumers live with the low quality of nutritional information on UPF, which is an obstacle for consumers to make healthier food choices^(49)^. Recently, the implementation of frontal labelling has been approved, but the industry has until the end of 2025 to adapt its packaging^(12,49)^.

The increase in the presence of FV advertising may help consumers make healthy food purchasing decisions. The use of printed materials, signs and food demonstrations to promote fresh foods are considered low-cost and feasible strategies^(50)^. In a research conducted in US grocery stores, exposure to FV advertising was able to influence the consumers’ food choices, especially when linked to monetary resources^(50)^.

Price is one of the main characteristics that can influence the purchase of food^(37)^. In the PAS area, a small increase in the UPF price index was observed, but its real impact on the consumer purchasing power needs to be further evaluated in future studies. The analysis of the variation in food prices in Brazil showed that, since the beginning of the 2000s, the price of UPF has undergone successive reductions^(51)^. Moreover, the forecast prices indicate that unhealthy food will become cheaper than healthy food in 2026^(49)^.

Regulatory policies, such as subsidies for the purchase of healthy foods and taxation of UPF, have been tested in different scenarios and are effective, but they are difficult to implement due to conflicts of interest with the food industry and agribusiness^(12,49)^. A successful example was the Mexican tax on sugar-sweetened beverages, which helped to increase the proportion of people who do not consume soda and reduce the proportion of people who consume large amounts^(13)^. In addition, such taxes can provide governments with additional resources to invest in health promotion actions^(12,49)^.

According to the HFSI, the access to healthy foods has increased but varied by type of store. Supermarkets increased access to healthy foods more than the FV market. Larger food retailers may find it easier to follow market trends. In contrast, the greater availability of UPF in small stores may be the result of expanding the supply of products to attract consumers and competing with larger food retailers that offer a wide variety of foods. The poorer access to healthy foods in the FV market is a major concern, as FV markets are the main place where PAS users can purchase fresh food. The increase in the availability of UPF, although NS, at the same time as a substantial increase in the advertising of these foods, may be impact on HFSI. This result possibly shows how much UPF has grown in the market and can compete with fresh foods^(35)^. Reducing access to healthy foods in these stores can expand or create areas with little healthy food supply^(6)^. Furthermore, this result shows the importance of considering the characteristics of the consumer environment in the investigation of the food environment. Analysis of the density or number of stores and open-air food markets may indicate a stability of the food environment^(52)^, but changes in the consumers’ food environment analysed by HFSI may indicate an increase in access to healthy food.

This study has some limitations that should be taken into account. First, the study analysed only the consumers’ food environment for home consumption and that sells FV. Further research is needed to assess changes in other types of stores, such as fast food and restaurants. It is worth mentioning that eating at home is still predominant in Brazil^(53)^, and therefore, this analysis is of great relevance.

In this study, an Euclidean distance was used to define the food environment, which is considered suitable for define the food environment analysis territory^(25)^. However, it is known that the vulnerability of the territory could impact differences in the food environment^(52)^. In this study, we chose not to adjust the data using HVI, as we wanted to observe the real change in the environment and the effect of vulnerability could not be nullified.

Despite the complexity of assessing the food environment given the variety of items that vary by type of stores and the objectivity involved in the evaluation, this study used a questionnaire with high reliability and adopted procedures^(29)^ to increase the reliability of the data, for example: being applied by trained food and nutrition professionals, using protocols to minimise errors and bias and carrying out practical training. In addition, we limited the study to assessing the presence of UPF advertising only; further studies are needed that consider the frequency and type of advertising in the store. Finally, the price analysis did not consider variations triggered by the market or changes in inflation during the period, which faced global economic crisis.

This is the first study in Brazil to assess changes in the food consumer environment. In addition, investigated areas of health promotion services from PHC, in line with public health policies. Research and investment in the promotion of healthy foods in health service territories, where a large circulation of people can be observed, may well be an important strategy for building healthy environments. However, the variability of the food environment requires caution when extrapolating the data to other scenarios, such as high-income countries.

The results showed just how essential it is to control UPF advertising, especially in places that sell fresh food. Governments urgently need to prioritise regulatory measures for advertising. Finally, we suggest that it is important to support the qualification of traders to encourage the sale of healthy foods, focusing on actions geared towards the characteristics of the consumer environment. In this sense, aspects that promote the quality of fresh food can be worked on, such as adequate storage and handling, arrangement of fresh products that favour purchase, qualification for purchase and supply control in order to avoid waste and guarantee variety for the consumer and the use of food advertising^(10)^.

## Conclusion

This study examined changes in the consumers’ food environment over a 5-year period in the health promotion service areas of Brazilian PHC in Belo Horizonte. The results showed important changes in the consumers’ food environment that may favour the promotion of healthy diets, with greater diversity, variety, quality and advertising of fresh foods. However, concomitantly, the increase in availability and advertising of UPF (in stores that sell FV) may reinforce an unfavourable competition with healthy foods. The changes in the consumers’ food environment, observed in the reduction of the HFSI, have also disfavoured FV markets, which are the main places where fresh foods can be purchased by the vulnerable population in Brazil. These unequal changes in all food stores types demonstrate the importance of food supply policies that promote a healthy environment and favour the maintenance of traditional healthy food retailers.

## Supporting information

de Freitas et al. supplementary material 1 de Freitas et al. supplementary material

de Freitas et al. supplementary material 2 de Freitas et al. supplementary material

## Footnotes

1 The HVI was developed by the Municipal Health Secretary of Belo Horizonte to guide the planning of health actions, including the PAS implementation site. The HVI combines socio-economic and sanitation variables using the scale of analysis of the census sector obtained in Brazilian demographic census. The HVI is categorised into four classes: low, medium, high and very high risk, in order to identify priority areas for intervention and a more effective resource allocation. In areas with a high HVI, the population presents an increased risk of negative health outcomes; and in areas with a medium HVI, the population’s health risk is similar to the average for municipality. In contrast, the population in low HVI areas has a low risk of negative health outcomes related to socioeconomic and sanitary conditions^(23)^.

## Supplementary material

For supplementary material accompanying this paper, visit https://doi.org/10.1017/S1368980024001721.
