# Supplementary material for: Consumer food environments change over a 5-year period
Source: Public Health Nutr. 2024 Oct 3;27(1):e187. doi: 10.1017/S1368980024001721 (PMC11505132; doi:10.1017/S1368980024001721)
Supplement: de Freitas et al. supplementary material 1 — de Freitas et al. supplementary material [file S1368980024001721sup001.docx]

**Supplementary Material 1**

Figure 1: Research sampling process

PAS units in the municipally (n=63)

Exclusion (n=13)

- Night operation (n=3)

- Closed before data collection (n=1)

- Located in areas with low HVI (n=7)

- Previous performance of nutrition interventions studies (n=2)

Eligible PAS units (n=50)

Preparation for randomization (separation of units according administrative district)^1^

Draw for the first unit in each administrative district (n=9)

Identification of HVI of the draw unit (n=9)

Draw for the second unit in each administrative district^2^ (n=9)

Note: HVI=health vulnerability index.

^1^Barreiro (medium HVI=5; hight HVI=3); Central-South (medium HVI = 3; high HVI=1); East (medium HVI=3); Northeast (medium HVI=3; high HVI=2); Northwest (medium HVI=6; high HVI=1); North (medium HVI=2; high HVI=6); West (medium HVI=2; high HVI=1); Pampulha (medium HVI=2; high HVI=2); Venda Nova (medium HVI=5; high HVI=2).^2^If the second unit drawn from the administrative district did not present an HVI similar to the first unit drawn, a new draw was carried out until a similar HVI was obtained.

Figure 2 - Distribution of PAS units by administrative district.

Belo Horizonte, 2012.


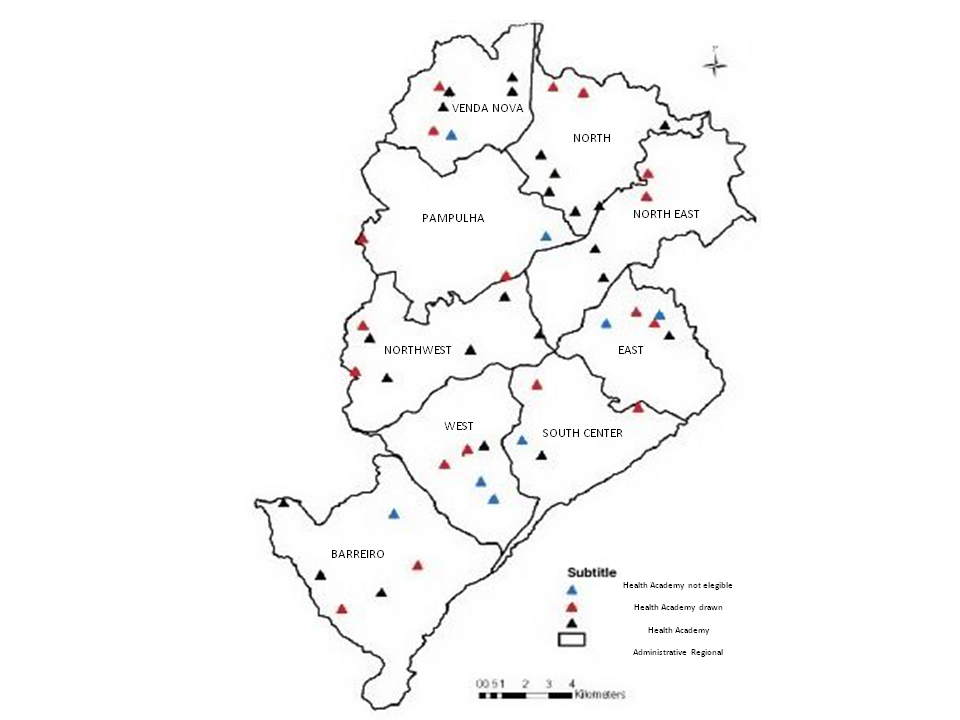


PAS units elegible, but not drawn

PAS units exclused

PAS units drawn

Administrative districts

Note: PAS – Programa Academia da Saúde

Data: Costa BV (2015). Alimentação e ambiente alimentar no território do programa Academia da Saúde de Belo Horizonte, Minas Gerais. PhD Thesis, Universidade Federal de Minas Gerais.
