# Supplementary material for: Consumer food environments change over a 5-year period
Source: Public Health Nutr. 2024 Oct 3;27(1):e187. doi: 10.1017/S1368980024001721 (PMC11505132; doi:10.1017/S1368980024001721)
Supplement: de Freitas et al. supplementary material 2 — de Freitas et al. supplementary material [file S1368980024001721sup002.docx]

**Supplementary Material** **2 - Scoring system for the Healthy Retail Food Store Index (HFSI).**

| **Variable** | **Score** |
| --- | --- |
| Fresh fruits and vegetable availability | 0 points if not available; 1 point if available |
| Fresh fruits and vegetable located near the entrance of the store | 0 points if not located near the entrance of the store; 1 point if located |
| Fruit diversity | 0 points if not available; 1 point if 1–7 types of the 10 most purchased fruits are available; 2 points if 8–10 of the 10 most purchased fruits are available |
| Fruits Variety | 0 points if not even 1 fruit variety is available; 1 point if up to 14 varieties are available; 2 points if 15 or more varieties are available |
| Vegetables diversity | 0 points if not available; 1 point if 1–7 types of the 10 most purchased vegetables are available; 2 points if 8–10 of the 10 most purchased vegetables are available |
| Vegetables variety | 0 points if not even 1 vegetable variety is available; 1 point if up to 14 varieties are available; 2 points if 15 or more varieties are available |
| Fruits and vegetable advertising | 0 points if not available; 1 point if available |
| Soft drinks availability | 0 points if available; 1 point if not available |
| Sugar-sweetened nectar/juice availability | 0 points if available; 1 point if not available |
| Chocolate filled cookies availability | 0 points if available; 1 point if not available |
| Corn chips availability | 0 points if available; 1 point if not available |
| Ultraprocessed foods advertising | 0 points if available; 1 point if not available |

Source: Duran AC, Diez Roux AV, Latorre Mdo R, Jaime PC. Neighborhood socioeconomic characteristics and differences in the availability of healthy food stores and restaurants in Sao Paulo, Brazil. Health Place. 2013 Sep; 23:39-47. doi: 10.1016/j.healthplace.2013.05.001. Epub 2013 May 18. PMID: 23747923; PMCID: PMC3758426.
